# Rhizomes as Multi-Target Pharmacological Platforms Against Tauopathy: Neuro-Metabolic Crosstalk, Drug-Likeness, and Translational Challenges

**DOI:** 10.3390/ph19050792

**Published:** 2026-05-19

**Authors:** Andreas Wilson Setiawan, Jinwon Choi, Sohyun Park, Min Choi, Raymond Rubianto Tjandrawinata, Edwin Hadinata, Moon Nyeo Park, Taruna Ikrar, Fahrul Nurkolis, Bonglee Kim

**Affiliations:** 1 Faculty of Medicine, Universitas Dian Nuswantoro, Semarang 50131, Indonesia; 2 Department of Pathology, College of Korean Medicine, Kyung Hee University, Seoul 02447, Republic of Korea; 3 School of Bioscience, Innovation and Technology, Atma Jaya Catholic University of Indonesia, Jakarta 12930, Indonesia; 4 School of Medicine, Faculty of Medicine, Ciputra University of Surabaya, Surabaya 60219, Indonesia; 5 The Indonesian Food and Drug Authority (BPOM), Jakarta 10560, Indonesia; 6 Faculty of Medicine, Universitas Airlangga, Surabaya 60131, Indonesia; fahrul.nurkolis.mail@gmail.com; 7 Institute for Research and Community Service, State Islamic University of Sunan Kalijaga (UIN Sunan Kalijaga), Yogyakarta 55281, Indonesia; 8 Medical Research Center of Indonesia, Surabaya 60281, Indonesia; 9 Korean Medicine-Based Drug Repositioning Cancer Research Center, College of Korean Medicine, Kyung Hee University, Seoul 02447, Republic of Korea

**Keywords:** tauopathy, rhizomes, multi-target pharmacology, neuro-metabolic crosstalk, CNS drug-likeness

## Abstract

Tauopathies, including Alzheimer’s disease (AD), progressive supranuclear palsy (PSP), corticobasal degeneration (CBD), and frontotemporal lobar degeneration with tau pathology, are unified by pathogenic tau misfolding, post-translational modification, aggregation, and network-level spread. Yet decades of drug development that predominantly pursued single nodes (e.g., one kinase, one aggregation inhibitor, one monoclonal antibody epitope) have repeatedly delivered late-stage disappointments, underscoring a central lesson: tauopathy behaves less like a linear pathway and more like a coupled system of proteostasis failure, neuroinflammation, synaptic-mitochondrial stress, and metabolic dysregulation. This review examines rhizomes (notably *Zingiberaceae* genera such as *Curcuma*, *Zingiber*, *Alpinia*, *Kaempferia*, and *Boesenbergia*) as chemically diverse “multi-target platforms” whose bioactives can engage several tau-relevant nodes simultaneously. We synthesise evidence across tau phosphorylation (GSK-3β/CDK5 and upstream stress signalling), tau aggregation and seeding, autophagy-lysosome and proteasome pathways, redox-mitochondrial resilience, neuroinflammatory circuits (NF-κB/NLRP3), and neuro-metabolic signalling (insulin-PI3K-AKT, AMPK-mTOR). A translational lens is applied throughout, focusing on drug-likeness and CNS multiparameter optimisation; BBB permeability and efflux; metabolism and bioavailability constraints; and formulation strategies (nanoparticles, phytosomes, engineered exosomes) that may render rhizome-derived scaffolds more clinically plausible. We conclude that rhizomes offer credible mechanistic hypotheses for tau modulation, but progress depends on rigorous standardisation, realistic exposure matching, biomarker-driven study design, and a shift from “single-compound optimism” to network pharmacology with translational discipline.

## 1. Introduction

Dementia is a growing global health burden, with tens of millions of people affected worldwide, and its prevalence projected to rise as populations age. The World Health Organization highlights dementia as a major public health issue, and Alzheimer’s Disease International regularly synthesises its global prevalence and economic impact [1,2,3]. In the United States, the Alzheimer’s Association reports millions living with Alzheimer’s dementia and anticipates substantial growth in the coming decades [4,5].

Within the broad dementia umbrella, tauopathy warrants special focus because tau pathology (hyperphosphorylated tau accumulation, tau oligomer “seeds”, and neurofibrillary tangles) tracks closely with neurodegeneration and clinical decline in Alzheimer’s disease (AD) and also defines primary tauopathy such as progressive supranuclear palsy (PSP) and corticobasal degeneration (CBD) [6,7,8]. Epidemiological estimates vary by diagnostic criteria and registry design, but PSP and CBD remain rare relative to AD, creating a second translational challenge: Primary tauopathy trials are difficult to power, while AD trials face heterogeneity and mixed pathologies [9].

A third challenge, and the one most relevant to pharmacology, is that single-target strategies have repeatedly struggled. In AD, phase 2 data for an anti-tau monoclonal antibody (tilavonemab) showed no meaningful clinical benefit versus placebo over 96 weeks despite acceptable tolerability [6,10]. Similarly, a phase 2 trial of the anti-tau antibody gosuranemab demonstrated robust target engagement (large reductions in unbound N-terminal tau in cerebrospinal fluid or CSF) without slowing clinical progression or altering tau positron emission tomography (PET) trajectory in target regions [11]. Even small-molecule strategies aimed directly at tau aggregation have stumbled; in a major phase 3 trial, the tau aggregation inhibitor leuco-methylthioninium bis(hydromethanesulfonate) (LMTM) did not show benefit for co-primary clinical outcomes as an add-on therapy [12]. These outcomes do not invalidate tau as a target; instead, they argue that tauopathy is a network pathology where upstream metabolic stress, inflammatory amplification, and impaired clearance can sustain tau dysfunction even if one molecular interaction is blocked [13].

This review is built around the idea that rhizomes are multi-target pharmacological platforms at the neuro-metabolic interface. Rhizomes are not “one molecule, one receptor” interventions; they are reservoirs of structurally diverse phytochemicals (curcuminoids, gingerols/shogaols, diarylheptanoids, sesquiterpenes, flavonoids) that can, in principle, modulate several tau-relevant nodes, kinase signalling, oxidative stress, neuroinflammation, neurobehavior [14], and proteostasis while also engaging systemic metabolic pathways (insulin sensitivity, lipid handling, mitochondrial function) [15]. The key novelty lies not in the general idea that rhizomes may benefit AD, but in a more focused proposition: tau pathology is actively shaped by neuro-metabolic crosstalk, including insulin resistance, AMPK–mTOR imbalance, ceramide signaling, and mitochondrial stress–inflammation loops. Within this framework, rhizome-derived compounds present plausible opportunities to act on multiple nodes of this interconnected system. However, this potential can only be meaningfully realized if critical factors such as drug-likeness, bioavailability, tissue exposure, standardization of extracts, and appropriate biomarker selection are treated as central scientific priorities rather than secondary considerations [16].

## 2. Tau Pathology as a Pharmacological Network

**Figure 1:**
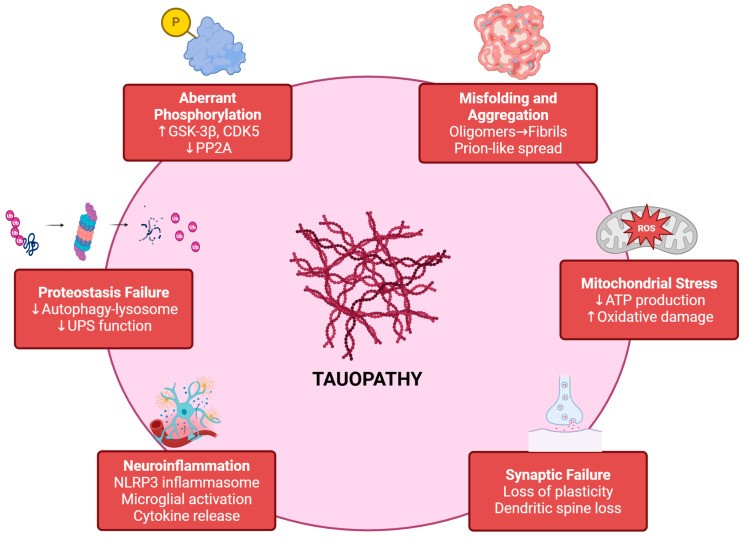
Tauopathy is a multi-node network disorder. Tau pathology emerges from interconnected mechanisms, including aberrant phosphorylation (GSK-3β, CDK5), misfolding and aggregation, impaired proteostasis (autophagy–lysosome and UPS dysfunction), mitochondrial stress, synaptic failure, and neuroinflammation. This network architecture explains the limited success of single-target interventions and provides a rationale for multi-target pharmacological strategies. Created in BioRender. Nurkolis, F. (2026) https://BioRender.com/rteq5i5.

Tau is a microtubule-associated protein encoded by microtubule-associated protein tau (MAPT), which supports axonal microtubule stability and contributes to neuronal structure and trafficking (Figure 1). In the adult human brain, multiple tau isoforms arise from alternative splicing, and the balance of isoforms and post-translational modifications influences microtubule binding and intracellular localisation [17,18,19]. Tauopathy emerges when tau undergoes pathological transitions: aberrant phosphorylation, conformational change, oligomerisation, fibril formation, and eventual accumulation as tangles and glial inclusions [7,13].

From a drug-discovery standpoint, the most useful way to frame tau biology is as a set of pharmacological nodes, places where intervention can, in principle, bend the trajectory of the system.

### 2.1. Phosphorylation and Signalling Balance (Kinases and Phosphatases)

Hyperphosphorylation is among the most prominent tau modifications in disease, and multiple kinases can phosphorylate tau at disease-associated epitopes. Reviews consistently emphasise the centrality of kinases such as GSK-3β and CDK5 (with contributions from MAPKs and MARK family kinases), while phosphatases, especially PP2A, constitute the major opposing force [13]. Importantly, these enzymes sit in broader signalling webs (PI3K-AKT, stress kinases, insulin signalling), which is why “kinase inhibition” is rarely a clean, one-node action in vivo [16].

### 2.2. Misfolding, Oligomers, and Aggregation

Tau aggregation is not merely a late-stage epiphenomenon; oligomeric tau species can be especially potent seeds for templated conversion, and interventions that prevent oligomer formation or reduce seed competence may be more valuable than those that only dissolve end-stage fibrils [20,21]. This framing helps reconcile why some “aggregation inhibitors” show biochemical effects yet fail clinically; the wrong tau species, wrong compartment, or wrong disease stage may be targeted [12,22,23].

### 2.3. Propagation and Cell-to-Cell Spread

A substantial body of work supports prion-like features of tau assemblies, including seeding and regional spread. While mechanistic details differ across models, this concept has been influential in motivating antibody strategies aimed at extracellular tau and spread blockade [24,25]. The disappointing outcomes of several anti-tau antibody trials suggest that blocking putative extracellular steps alone may be insufficient once intracellular proteostasis and neuroinflammatory amplification are entrenched [6,26].

### 2.4. Proteostasis Failure: UPS and Autophagy-Lysosome Pathway

Tau clearance depends on coordinated protein quality control pathways. Ubiquitin proteasome function and autophagy lysosome flux can both influence tau accumulation, and lysosomal regulators such as transcription factor EB (TFEB) have emerged as promising “master switches” for enhancing clearance capacity [27]. The implication for multi-target phytochemicals is straightforward: if a compound modestly reduces tau phosphorylation but also enhances autophagy or proteasome activity, the combined effect may be larger than either action alone [28,29].

### 2.5. Neuroinflammation as a Tau Amplifier

Microglia and inflammasome pathways are deeply interwoven with tau pathology and neurodegeneration, but the causal direction can be bidirectional. Notably, recent mechanistic work suggests tau can directly promote NLR family pyrin domain-containing 3 (NLRP3) inflammasome activation (including via NLRP3 acetylation), providing a direct biochemical bridge between tau species and innate immune amplification [30,31]. At the same time, some studies indicate that simply deleting inflammasome components does not automatically rescue tau pathology in certain tau mouse models, warning against overly linear “inflammation causes tau” narratives [1].

Taken together, tauopathy is best understood as a networked process in which phosphorylation dynamics, seeding competence, propagation, clearance efficiency, inflammation, and metabolic stress continuously interact and reinforce one another. This systems-level perspective frames the central focus of this review: whether rhizome-derived compounds can achieve meaningful multi-node modulation that reflects the biological complexity of tauopathy and whether such effects can be translated with credible pharmacokinetic profiles and robust biomarker strategies.

## 3. Neuro-Metabolic Crosstalk as a Therapeutic Leverage Point

**Figure 2:**
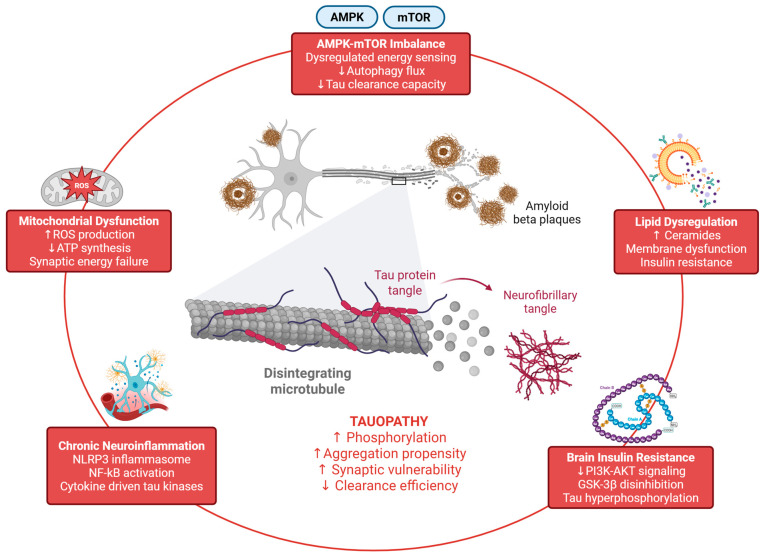
Neuro-metabolic mechanisms influencing tau pathology. Brain insulin resistance, AMPK–mTOR imbalance, mitochondrial dysfunction, lipid dysregulation, and chronic neuroinflammation collectively modulate tau phosphorylation, aggregation propensity, and clearance efficiency. These processes define the neuro-metabolic interface as a key therapeutic leverage point. Created in BioRender. Nurkolis, F. (2026) https://BioRender.com/64oazm2.

The “neuro-metabolic interface” is not a metaphor; it is a mechanistic reality in which peripheral metabolic dysfunction and brain energy signalling can shape tau phosphorylation, clearance, and vulnerability (Figure 2). Evidence from human studies, animal models, and molecular pathway analyses supports the view that insulin resistance, lipid dysregulation, mitochondrial stress, and chronic inflammation increase susceptibility to tau pathology and accelerate cognitive decline [16,32].

### 3.1. Brain Insulin Resistance and PI3K-AKT-GSK-3β Coupling

A key molecular bridge is the insulin/IGF signalling pathway; impaired insulin signalling reduces AKT activity, which can disinhibit GSK-3β, thereby favouring tau hyperphosphorylation. Reviews of insulin resistance in AD repeatedly emphasise overlapping mechanisms that include altered PI3K-AKT signalling, oxidative stress, and neuroinflammation [16,33]. Importantly, insulin signalling also intersects with synaptic plasticity and mitochondrial function, meaning that the downstream consequence is not only tau phosphorylation but also broader synaptic fragility [4,33].

### 3.2. AMPK-mTOR-Autophagy Axis: Energy Sensing Meets Clearance

AMPK and mTOR are canonical regulators of cellular energy and autophagy; however, their relationship is more nuanced than older “AMPK always activates autophagy” frameworks. Recent mechanistic work has shown context-dependent effects, including situations where AMPK phosphorylation of ULK1 can inhibit rapid autophagy induction [34,35]. For tauopathy, the practical point is that energy stress and autophagy regulation are tightly coupled, and interventions that shift this balance could change tau clearance capacity, especially if they also engage TFEB-driven lysosomal biogenesis [27].

### 3.3. Mitochondria, ROS, and Synaptic Failure

Tau can associate with synaptic mitochondria and disrupt mitochondrial function, which can contribute to synaptic loss and neuronal vulnerability [36,37]. This creates a self-reinforcing loop, with mitochondrial dysfunction increasing ROS and energetic deficits, which can activate stress signalling and inflammation, potentially promoting further tau modification and impaired clearance [4,38].

### 3.4. Neuroinflammation and Inflammasome Circuits

Microglial activation is an early and persistent feature of AD and many tauopathies, with pathways such as NF-κB and inflammasome activation shaping cytokine environments and synaptic pruning [39,40,41]. The emerging observation that tau species can directly engage inflammasome activation mechanisms suggests that tau and inflammation may be coupled not only indirectly (via damage signals) but also through more direct molecular interactions [30,42].

### 3.5. Lipid Dysregulation, Ceramides, and Membrane Biology

Lipid homeostasis influences membrane microdomains, receptor signalling, and inflammatory tone. Ceramides have been advanced as key mediators that can aggravate brain insulin resistance and contribute to Aβ and tau pathology, partly by promoting inflammatory and oxidative stress pathways [43]. This is a particularly relevant interface for rhizomes because many rhizome constituents show systemic metabolic effects (altered glucose handling, lipid profiles, adipokines), raising the possibility that peripheral metabolic modulation can translate into central tau-relevant shifts if the CNS exposure is adequate and sustained [44].

The neuro-metabolic perspective helps clarify why multi-target strategies are often more rational in addressing complex diseases. In reality, tau pathology does not occur in isolation; it is embedded within a broader context of disrupted energy metabolism, chronic immune activation, and impaired clearance systems [45]. Therefore, any candidate therapy, whether derived from natural products or not, should be evaluated based on its capacity to modulate this interconnected system as a whole, rather than simply demonstrating the ability to bind a single protein in vitro [6].

## 4. Rhizomes as Multi-Target Platforms Against Tauopathy

**Figure 3:**
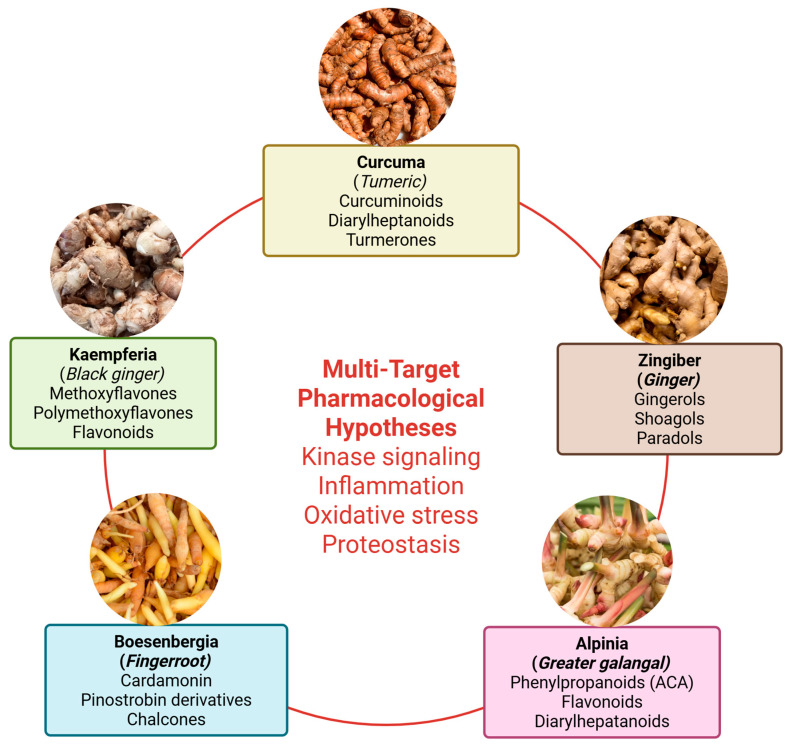
Major rhizome genera relevant to neuropharmacology. Representative rhizomes, including Curcuma, Zingiber, Alpinia, Boesenbergia, and Kaempferia, provide chemically diverse phytoconstituents such as curcuminoids, gingerols, shogaols, flavonoids, and phenylpropanoids, forming the basis of multi-target pharmacological hypotheses. Created in BioRender. Nurkolis, F. (2026) https://BioRender.com/amlatdw.

Rhizomes, especially from the Zingiberaceae family, are a practical and scientifically interesting entry point for multi-target pharmacology because they combine ethnopharmacological plausibility (longstanding dietary and medicinal use), chemical diversity, and a growing body of mechanistic work touching kinase signalling, inflammation, oxidative stress, and proteostasis (Figure 3) [46].

Key rhizome genera often discussed in neuropharmacology include *Curcuma* (turmeric species), *Zingiber* (ginger), *Alpinia* (galangal relatives), *Kaempferia* (e.g., black ginger), and *Boesenbergia* (fingerroot). Each contains different dominant chemical classes: curcuminoids and turmerones (in *Curcuma*), gingerols/shogaols (in *Zingiber*), phenylpropanoids like 1′-acetoxychavicol acetate (ACA) (in *Alpinia*), methoxyflavones (in *Kaempferia*), and diverse flavonoids such as cardamonin/pinostrobin derivatives (in *Boesenbergia*) [46,47].

A recurring translational obstacle is standardisation. Rhizomes vary by cultivar, geography, harvest time, and processing (drying, heating, extraction solvent). For ginger, a well-established example is the conversion of gingerols into shogaols during thermal processing or storage, meaning that an “extract” is not a fixed entity unless processing is controlled and marker compounds are quantified [48,49]. For turmeric, essential oil composition and curcuminoid profiles can vary by variety and conditions, complicating reproducibility unless chemotyping and quality control are built into experimental designs [50,51].

The most convincing evidence that rhizome-derived compounds can influence tau pathology beyond general neuroprotection is largely centered on curcumin and its derivatives. These compounds demonstrate direct, tau-specific actions, including inhibition of tau aggregation, modulation of aberrant phosphorylation, and enhancement of tau clearance mechanisms [2]. In contrast, bioactive constituents from other rhizomes tend to exert their effects more indirectly. Their roles are primarily linked to improvements in metabolic regulation, attenuation of inflammation and oxidative stress, and maintenance of proteostasis, which may collectively contribute to mitigating tau-related neurodegeneration without directly targeting tau pathology itself [2].

An overview of metabolomic profiling, network pharmacology, and in silico predictions supporting tau-relevant mechanisms is summarised in Table 1. At present, the tau-focused evidence base remains heavily curcumin-dominant, particularly regarding direct effects on tau aggregation, phosphorylation, and clearance pathways. In contrast, most non-curcumin rhizome bioactives, including compounds derived from *Zingiber*, *Alpinia*, *Kaempferia*, and *Boesenbergia*, are supported primarily by indirect evidence related to oxidative stress, neuroinflammation, insulin signalling, mitochondrial protection, or broader neuro-metabolic modulation. While these mechanisms may still influence tau pathology at the systems level, direct validation using tau-specific endpoints, tauopathy models, and exposure-matched experimental designs remains comparatively limited.

Collectively, the studies summarised in Table 1 provide primarily hypothesis-generating and prioritisation-level evidence rather than definitive proof of anti-tau efficacy. Among the included compounds, curcumin currently has the strongest mechanistic relevance because several studies extend beyond general neuroprotection into tau-associated kinase signalling, metabolic regulation, and CNS exposure assessment. In contrast, evidence for *Zingiber*, *Boesenbergia*, *Kaempferia*, and *Alpinia*-derived compounds remains largely indirect, relying on network pharmacology, docking, metabolic signalling inference, or upstream disease-modifying pathways rather than direct interrogation of tau aggregation, seeding, phosphorylation, or clearance endpoints.

From a translational perspective, the table also highlights a major evidence gap between computational plausibility and biologically validated tau modulation. While in silico docking, ADMET prediction, and pathway mapping are valuable for compound prioritisation, they cannot establish meaningful disease modification without exposure-matched validation in tau-relevant cellular and in vivo systems. Overall, the current landscape can therefore be interpreted as curcumin-dominant with several emerging rhizome candidates that require substantially stronger tau-specific experimental validation.

## 5. Mechanisms and Multi-Target Pharmacology of Rhizome Bioactives Against Tau Pathology

**Figure 4:**
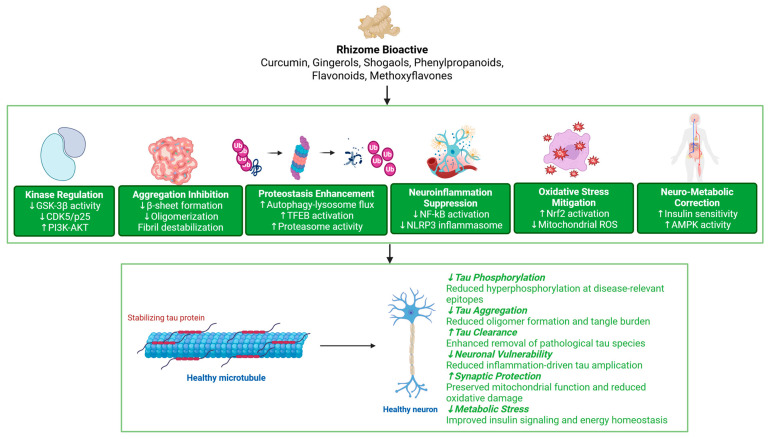
Multi-target pharmacological actions of rhizome-derived bioactives. Rhizome phytochemicals modulate tau-relevant mechanisms via kinase regulation, inhibition of aggregation, enhancement of proteostasis, suppression of neuroinflammation, mitigation of oxidative stress, and correction of neuro-metabolic dysfunction. Created in BioRender. Nurkolis, F. (2026) https://BioRender.com/p0uodxn.

A credible rhizome-to-tau argument must be mechanistic and integrative (Figure 4). The goal is not to compile isolated “curcumin did X” statements, but to map rhizome chemistry onto tau-relevant nodes while acknowledging when evidence is indirect, exposure is unrealistic, or endpoints are not truly tau-specific [2]. Key in vitro studies interrogating tau-related and upstream neuro-metabolic nodes are compiled in Table 2.

The in vitro evidence in Table 2 demonstrates important differences in evidential strength across rhizome bioactives. Curcumin and several curcumin-derived formulations provide the most direct tau-focused evidence, including modulation of tau phosphorylation, interference with tau aggregation, and effects on tau oligomer pathways in neuronal systems [2,59,60,61]. These findings move beyond general antioxidant or neuroprotective claims and directly interrogate molecular nodes central to tauopathy biology. By comparison, most non-curcumin rhizome compounds primarily influence upstream or parallel mechanisms such as oxidative stress reduction, AKT/GSK-3β signalling balance, proteasome activity, or neuronal resilience, often without measuring tau species directly.

Importantly, many cellular studies also operate at concentrations that may exceed realistic brain exposure levels achievable through conventional oral administration. As a result, several experiments should be interpreted primarily as mechanistic pathway probes rather than direct indicators of clinical feasibility. Therefore, the stronger translational studies are those integrating formulation strategies, pharmacokinetic considerations, or disease-relevant tau endpoints, particularly when multiple interconnected tau-related nodes are evaluated simultaneously.

### 5.1. Modulation of Tau Phosphorylation (GSK-3β/CDK5 and Upstream Stress Pathways)

Multiple lines of evidence support curcumin’s capacity to influence tau phosphorylation states in disease-relevant models. In a scopolamine-induced AD-like rat model, curcumin administration reduced active GSK-3β signalling and CDK5/p25-related changes and was associated with decreased tau phosphorylation at disease-relevant epitopes, alongside behavioural improvements [63]. At the cellular level, curcumin attenuated Aβ-induced tau hyperphosphorylation in SH-SY5Y neuroblastoma cells through a pathway involving PTEN/AKT/GSK-3β signalling [59,64]. Curcumin has also been reported to reduce abnormal tau phosphorylation under stress conditions (e.g., acrylamide-induced neurotoxicity) by suppressing PERK-eIF2α stress signalling with downstream effects on GSK-3β and tau phosphorylation [60,65].

### 5.2. Inhibition or Modulation of Tau Aggregation and Oligomer Pathways

Direct biophysical evidence indicates that curcumin can interfere with tau’s conversion into β-sheet-rich assemblies. A widely cited study reported that curcumin inhibited tau aggregation and could disintegrate preformed tau filaments in vitro, implying a potential role not only in prevention but also in disrupting existing aggregates under certain conditions [2,66]. Beyond curcumin itself, designed curcumin derivatives have been shown to modulate tau oligomer aggregation pathways in neuronal models, with the stated aim of reducing toxicity associated with oligomeric species [61,67].

### 5.3. Proteostasis Enhancement: Autophagy-Lysosome Pathway, TFEB, and the Proteasome

Tau clearance capacity is a major determinant of pathology progression. The TFEB axis is especially relevant: TFEB regulates lysosomal biogenesis and autophagic capacity, and experimental stimulation of TFEB-mediated programmes can promote pathological tau clearance in cellular and mouse models [27,68,69]. Curcumin-inspired strategies have moved beyond “free curcumin” into delivery and pathway-activating designs. For example, engineered exosome formulations of curcumin were reported to improve cognitive performance and reduce tau phosphorylation in tau transgenic mice, with a mechanistic emphasis on mitochondrial protection and reduced oxidative stress, an example of a formulation acting through both bioavailability and cellular mechanisms [53].

Proteasome modulation is another plausible rhizome-to-tau bridge. ACA, a phenylpropanoid found in *Alpinia* rhizomes, increased proteasome activity in differentiated PC12 cells and improved cell viability under amyloid-β fragment stress, with evidence pointing to cAMP/PKA involvement. While tau-specific endpoints were not the focus of that work, proteasome enhancement is conceptually relevant to tau clearance and to preventing the accumulation of misfolded proteins under stress [28,70].

### 5.4. Neuroinflammation Control (NF-κB/NLRP3 and Microglial State)

Rhizome bioactives are frequently reported to downshift inflammatory signalling, though the translational importance depends on whether this occurs at CNS-relevant concentrations and whether it impacts tau-specific inflammatory coupling. Microglia-focused reviews emphasise NF-κB-linked cytokine programmes and the relationship between immune activation and tau pathology progression [40]. If tau can directly promote inflammasome activation (as suggested in recent mechanistic studies), then anti-inflammatory rhizome compounds may reduce not only “general inflammation” but tau-amplifying loops, provided they reach the right compartment and stage [30].

### 5.5. Oxidative Stress and Mitochondria: Reducing Upstream Pressure on Tau

Oxidative stress can push tau biology toward phosphorylation, misfolding, and impaired clearance. In metabolic disease contexts that are relevant to tauopathy risk (e.g., diabetes-associated brain vulnerability), curcumin nanoparticles improved behavioural performance and reduced tau hyperphosphorylation in a combined type 2 diabetes and neurodegeneration rat model, alongside reductions in inflammatory cytokines and improvements in oxidative stress markers [71]. In a tau transgenic setting, engineered exosomal curcumin constructs were reported to reduce mitochondrial ROS, protect neurons from apoptosis, improve memory-related behaviours, and suppress tau phosphorylation more effectively than free curcumin in the same experimental series linking mitochondrial protection, oxidative stress reduction, and tau state [53].

### 5.6. Neuro-Metabolic Pharmacology: Insulin Sensitisation and Energy Metabolism

Several rhizome constituents show systemic metabolic effects that may become centrally relevant by shifting insulin resistance and lipid signalling pressures that feed tau pathology. A ginger study in a brain insulin resistance model (intracerebroventricular streptozotocin) reported changes in molecular markers, including insulin receptor substrate (IRS) expression and GSK-3β gene expression, consistent with the idea that ginger can act within the insulin GSK-3β space [54,72]. In curcumin-focused work, improvements in insulin signalling and energy metabolism have been reported in AD-relevant models, supporting a mechanistic bridge whereby improved metabolic signalling reduces tau-phosphorylating pressures [52].

## 6. Drug-Likeness, Delivery, and Translational Hurdles

**Figure 5:**
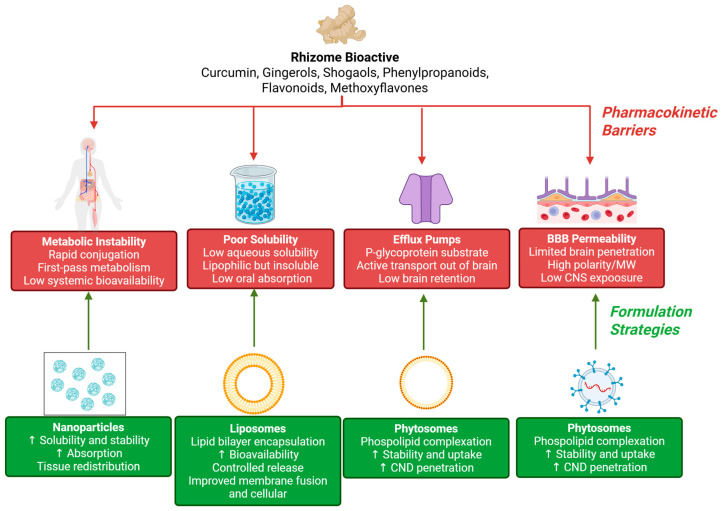
Drug-likeness and pharmacokinetic constraints for rhizome bioactives. Many rhizome phytochemicals face limitations related to solubility, metabolic stability, blood–brain barrier (BBB) permeability, and efflux. Translational feasibility increasingly depends on formulation strategies, including nanoparticles, liposomes, phytosomes, and exosome-based delivery. Created in BioRender. Nurkolis, F. (2026) https://BioRender.com/185pa05.

The gap between “promising mechanisms” and “clinical tauopathy therapy” is often pharmacokinetic. Rhizome bioactives mostly evolved for plant ecology, not human pharmacology, and many fail classical property filters for oral bioavailability, stability, and consistent CNS exposure (Figure 5) [73,74].

### 6.1. Physicochemical Constraints and Drug-Likeness Frameworks

Lipinski’s “rule of five” and related heuristic rules were built to anticipate absorption and permeability challenges in small molecules; Veber’s emphasis on polar surface area and rotatable bonds further highlights how high polarity or flexibility can impair oral bioavailability [75]. Because tauopathy therapies must often reach the brain, CNS-specific property optimisation becomes relevant. The CNS multiparameter optimisation (CNS MPO) framework was designed to help prioritise compounds more likely to cross the BBB while maintaining a safer property space [76,77]. Many polyphenols and bulky phytochemicals sit in an awkward zone: lipophilic enough to partition into membranes, but too insoluble, too metabolically labile, or too easily effluxed to sustain meaningful brain concentrations [73,78].

### 6.2. BBB Permeability and Efflux Complexities

BBB penetration is not a single parameter; it is shaped by polarity, ionisation, transporter interactions, and plasma protein binding. Even when a molecule can enter the brain, it may not distribute into the intracellular compartments where tau aggregation and phosphorylation cascades occur [76,79].

### 6.3. Metabolism and Bioavailability: The Curcumin Case Study

Curcumin is a paradigmatic example of the “potent in vitro, difficult in vivo” problem. In a controlled clinical trial in AD, plasma levels of native curcumin were low, and no clinical or biomarker efficacy was demonstrated, even with gram-level daily dosing, an outcome the authors explicitly linked to limited bioavailability [73,80]. Analytical work confirming the detectability of curcumin metabolites in serum and urine highlights that metabolism (including conjugation) is a central determinant of exposure and may matter as much as receptor binding [81].

### 6.4. Formulation Strategies: From “Nutraceutical” to Delivery-Engineered Candidate

The most credible path forward for rhizome-derived anti-tau strategies may be formulation-driven rather than raw-extract-driven. Several approaches now appear repeatedly in preclinical work:

1. Nanoparticle formulations have been shown to improve stability and absorption and to reshape tissue distribution. In a diabetes-associated neurotoxicity model, curcumin nanoparticles at specific doses reduced tau hyperphosphorylation and improved behavioural outcomes more robustly than some comparator regimens, illustrating how formulation can change effect sizes [71].
2. Engineered exosomes can act as delivery vehicles that target neuronal mitochondria and improve brain distribution profiles; in tau transgenic mice, exosome-curcumin constructs were associated with improved cognition and reduced tau phosphorylation [53].
3. Phytosomes and other lipid-based carriers for gingerols/shogaols can support stability and cellular uptake in neurotoxicity paradigms, representing a transferable strategy for rhizome bioactives beyond curcumin [58].

These strategies align with a sobering but productive translational principle: if a compound cannot achieve a plausible brain exposure window, pathway-aligned mechanisms will not matter clinically [76].

### 6.5. Preclinical Model Limitations and Dose Realism

Another recurring translational weakness is concentration mismatch. For example, 6-gingerol showed protective effects in a PC12 Aβ challenge model at concentrations of tens to hundreds of micromolar [62]. Such concentrations can be challenging to attain in brain tissue through dietary or oral supplementation, especially without advanced formulations. This does not invalidate mechanistic value, but it changes how results should be interpreted. Many studies function as pathway probes (what could happen if the node is modulated) rather than as direct evidence that a simple extract will meaningfully treat human tauopathy [76].

### 6.6. Evidence Hygiene: Retractions and Reproducibility

Botanical and natural product research is not immune to retractions. A notable example in the turmeric/AD space is a transgenic mouse study reporting substantial reductions in phosphorylated tau after an “optimised turmeric extract”, which later received a retraction notice [82]. For a translational review, this matters less as a historical detail and more as a reminder that extraction variability, selective reporting, and over-interpretation can accumulate quickly in complex interventions.

### 6.7. Publication Bias, Negative Findings, and Reproducibility Limitations

A balanced interpretation of the rhizome literature also requires acknowledging the substantial risk of publication bias and reproducibility limitations within the broader natural product and neurodegeneration fields. Positive findings involving antioxidant, anti-inflammatory, or cognitive effects are more likely to be published than null or contradictory results, particularly in small preclinical studies with heterogeneous extraction methods, variable formulations, and inconsistent endpoint selection. This creates a literature environment in which apparent mechanistic convergence may partly reflect selective reporting rather than true reproducible biological consistency.

Importantly, several clinically informative studies have produced mixed or negative outcomes despite promising preclinical rationale. Curcumin trials in Alzheimer’s disease, for example, demonstrated limited biomarker or clinical efficacy despite acceptable tolerability and extensive mechanistic justification, highlighting the persistent gap between in vitro activity and meaningful therapeutic exposure in humans. Likewise, failures of anti-tau antibodies and tau aggregation inhibitors reinforce the broader translational difficulty of modifying tauopathy progression even when target engagement is achieved.

Reproducibility is further complicated by variability in rhizome composition across cultivars, processing conditions, extraction solvents, and formulation technologies. Without rigorous chemical standardisation and exposure verification, studies nominally investigating the same “extract” may in fact evaluate substantially different phytochemical mixtures. Together, these issues underscore the need for exposure-matched experimental design, transparent reporting standards, publication of negative findings, and biomarker-driven validation pipelines to improve evidence reliability in future rhizome-based tauopathy research.

### 6.8. Clinical Biomarker Strategy: Aligning Rhizome Trials with Modern Tau Readouts

Tau-targeting drug development has improved scientifically (even amid failures) due to biomarker evolution, CSF and plasma phosphorylated tau measures, tau PET imaging, and coupled neurodegeneration readouts, which enable target engagement and disease staging with growing precision. Anti-tau antibody trials illustrate that gosuranemab produced strong CSF target engagement without clinical benefit, and tau PET substudies detected expected tau accumulation over time but no treatment effect [11,83,84]. This is exactly the level of clarity rhizome-derived interventions will need if they are to move beyond “cognitive supplement” narratives toward disease-modifying claims.

## 7. Evidence Across In Vivo Models and Human Studies

In vivo and clinical evidence are where rhizome-based hypotheses either become plausible or remain speculative. In general, curcumin and its derivatives dominate tau-specific in vivo evidence, while other rhizomes often contribute mechanistically relevant (but tau-indirect) signals [67]. Representative animal studies evaluating rhizome-derived interventions on tau pathology and neuro-metabolic coupling are summarised in Table 3.

The in vivo evidence presented in Table 3 further reinforces the current imbalance between curcumin-centered and non-curcumin rhizome evidence. Curcumin formulations, nanoparticles, and derivatives demonstrate the most robust tau-specific outcomes, including reductions in phosphorylated tau, modulation of tau-associated kinase signalling, and improvements in behavioural phenotypes in established tauopathy or neurodegeneration-related animal models. Several studies additionally incorporate mechanistic links to mitochondrial protection, oxidative stress reduction, or neuro-metabolic regulation, thereby supporting a systems-level interpretation of tau modulation.

In contrast, studies involving ginger, Kaempferia, or Alpinia-derived compounds predominantly provide indirect support through behavioural improvement, anti-inflammatory effects, insulin signalling modulation, or proteostasis-related mechanisms without direct quantification of tau pathology. While such upstream effects may still be biologically relevant to tauopathy progression, the absence of tau-specific endpoints limits the strength of causal inference. Consequently, the overall preclinical evidence remains strongest for curcumin-derived strategies, whereas other rhizome candidates should presently be regarded as mechanistically plausible but comparatively early-stage contributors to multi-target tauopathy research.

Human evidence is more limited and, unsurprisingly, more mixed, often because exposure constraints and population heterogeneity can overwhelm small effect sizes. Still, some studies are informative because they incorporate long-duration, bioavailable formulations and imaging or biomarker endpoints. Clinical investigations of rhizome-derived compounds and formulations relevant to cognition and tau-associated endpoints are outlined in Table 4.

From a translational standpoint, rhizome-derived compounds are currently positioned more credibly as preventive or adjunctive candidates rather than established disease-modifying therapies. Existing human studies predominantly involve cognitively normal individuals, older adults with mild cognitive concerns, or heterogeneous AD populations, with most trials focusing on cognitive performance, tolerability, or metabolic-inflammatory modulation rather than direct alteration of tau pathology progression. Consequently, the strongest near-term clinical rationale may lie in multimodal strategies where rhizome-derived interventions complement standard care or target upstream neuro-metabolic risk states such as insulin resistance, oxidative stress, and chronic inflammation. Definitive positioning as disease-modifying anti-tau therapies will require substantially stronger evidence integrating plasma or CSF p-tau measures, tau PET trajectories, exposure-confirmed pharmacokinetics, and durable functional outcomes in biomarker-characterised populations.

A small but important interpretive point: clinical studies in cognitively normal or mildly impaired adults that show cognitive benefits do not automatically imply anti-tau disease modification. However, such studies can still be valuable as tolerability and exposure pilots, pharmacodynamic readouts for inflammation/metabolic markers, and platforms to incorporate modern plasma p-tau measures and tau PET in future designs [93].

An adjacent translational lesson comes from the neuro-metabolic drug pipeline. Large trials of GLP-1 receptor agonists have been motivated partly by insulin-inflammation-neurodegeneration coupling. Yet the field is sobering: two phase 3 trials of oral semaglutide in early AD reported biomarker improvements without clinical superiority over CDR-SB, leading to discontinuation of the extension period [94]. Novo Nordisk’s semaglutide programme illustrates a broader principle relevant to rhizomes: metabolic and inflammatory biomarker shifts may not be sufficient unless they translate into durable cognitive/functional benefit and, ideally for tauopathy, demonstrable effects on tau biomarkers or tau PET trajectories [12,94].

## 8. Future Directions and Conclusions

**Figure 6:**
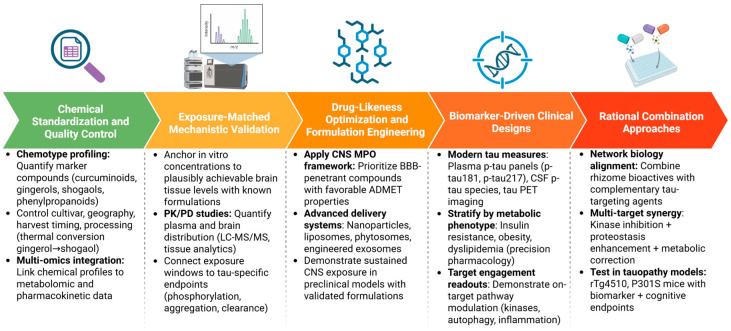
Translational roadmap for rhizome-derived tau-modulating strategies. Successful development requires chemical standardisation, exposure-matched validation, drug-likeness optimisation, biomarker-driven designs, and rational combination approaches aligned with tauopathy network biology. Created in BioRender. Nurkolis, F. (2026) https://BioRender.com/np2wcti.

Rhizomes can be defended as plausible multi-target platforms against tauopathy, but only under a translationally strict interpretation of the evidence (Figure 6). Direct anti-tau effects (aggregation inhibition, phosphorylation modulation, clearance enhancement) are best supported for curcumin and engineered curcumin derivatives or delivery systems in tauopathy mouse models (rTg4510, P301S) and using mechanistically anchored formulations (exosome-based constructs, nanoparticles) [2]. For other rhizomes such as ginger, galangal relatives, fingerroot, and black ginger, their strongest near-term value may be as network modulators that reduce upstream pressures on tau (insulin resistance signalling, oxidative stress, inflammation, proteostasis demand), while the field builds more tau-specific endpoints into experimental pipelines [54].

### 8.1. Precision Pharmacology at the Neuro-Metabolic Interface

Rather than broadly testing “rhizome extract X for AD,” more informative and mechanistically meaningful approaches would incorporate stratification based on metabolic phenotype—such as insulin resistance, obesity, and dyslipidaemia. These conditions are not merely comorbidities but active modulators of disease biology, as they significantly influence tau phosphorylation dynamics, proteostatic capacity, mitochondrial function, and neuroinflammatory tone [43]. Consequently, the effect of pathological tau accumulation and the efficiency of its clearance can differ substantially across these metabolic states. Integrating such stratification would enable a more precise evaluation of therapeutic responses, uncover phenotype-specific mechanisms of action, and ultimately improve the translational relevance of rhizome-derived interventions for tauopathies.

### 8.2. Exposure-Matched Mechanistic Validation

In vitro studies should increasingly be grounded in concentration ranges that are realistically achievable in brain tissue with well-characterized formulations. This alignment is essential to ensure that mechanistic insights are not built on exposure levels that cannot be attained in vivo [76,95]. By integrating pharmacokinetic considerations—such as absorption, distribution, blood–brain barrier penetration, and metabolic stability—early in experimental designs, researchers can generate findings that are more biologically and translationally meaningful [96,97,98], rather than relying on effects observed only under impractical or non-physiological conditions.

### 8.3. Multi-Omics and Metabolomics Integration

The most compelling rhizome-to-tau research will be integrative by design, linking chemical profiling (defining what is present in the extract), metabolomic and pharmacokinetic characterization (determining what actually reaches the circulation and brain), and pathway-level biomarker responses (identifying which biological nodes are modulated) within a single, coherent experimental framework [99,100]. Such alignment allows for a more credible attribution of biological effects to specific compounds or compound classes, reduces ambiguity in mechanism-of-action claims, and substantially strengthens the translational bridge from bench to potential clinical application [101].

### 8.4. Biomarker-Forward Clinical Designs

Human studies should integrate modern tau measures, plasma p-tau panels, CSF p-tau species, and tau PET where feasible so that null cognitive outcomes can be interpreted mechanistically rather than ambiguously [93,102]. Furthermore, rhizomes represent a credible pharmacological idea in tauopathies because tau pathology is embedded in neuro-metabolic dysfunction, and rhizome bioactives can, at least in principle, touch multiple nodes: kinase signalling, proteostasis, mitochondria/redox biology, and inflammation [63]. The field’s responsibility now is to treat rhizomes as pharmacological platforms rather than folkloric supplements, standardise their chemistry, quantify exposure, prioritise drug-likeness and delivery, and test hypotheses with modern tau biomarkers. Only then can rhizome-derived strategies be meaningfully evaluated as therapeutic opportunities to treat tau-driven neurodegeneration [73].

## Figures and Tables

**Table 1:** Metabolomic and in silico evidence linking rhizome bioactives to tau-relevant neuro-metabolic nodes.

Rhizome/Genus and Bioactive(s)	Evidence Type	Platform or End Point(s)	Tau-Related or Neuro-Metabolic Inference	Key Translational Note	Primary Source
*Curcuma* (curcumin)	Network pharmacology or docking	Integrated target prediction and pathway mapping for AD	Supports multi-target hypotheses consistent with kinase signalling, inflammation, and metabolic pathway modulation relevant to tau networks	Hypothesis-generating, requires exposure-matched validation	[52]
*Curcuma* (curcumin formulations)	PK + tissue distribution analytics (LC-MS/MS)	Quantification of curcumin distribution in plasma and brain tissue after IV administration	Enables realistic exposure discussion for central nervous system (CNS)-targeting strategies (a prerequisite for tau pathway engagement in vivo)	Links formulation to measurable brain delivery	[53]
*Curcuma* (curcumin)	Metabolic imaging (neuro-metabolic endpoint)	Functional readouts of cerebral glucose metabolism in transgenic AD models	Supports insulin energy pathway modulation upstream of tau phosphorylation (AKT-GSK-3β coupling)	Metabolic endpoints can be closer to clinical translation than some molecular assays	[52]
*Zingiber* (ginger extract)	Systems-level in vivo molecular profiling (metabolic signalling emphasis)	ICV-STZ brain insulin resistance model; gene expression shifts (IRS, BACE1, GSK-3β)	Positions ginger within insulin resistance → GSK-3β pathway space that can affect tau phosphorylation propensity	Indirect effects on tau unless p-tau/seed measures are added	[54]
*Boesenbergia* (boesenbergin A)	In silico ADMET + docking	Docking to human AChE; ADMET property predictions	Suggests CNS-feasible properties and mechanistic interaction with symptomatic target; indirect relevance to tau via network effects on cognition and neurotransmission	Useful for prioritisation, not proof of disease modification	[55]
*Boesenbergia* (cardamonin, pinocembrin, pinostrobin)	Enzyme kinetics + docking	BACE1 inhibition with docking, suggesting a non-competitive mode	Indirect tau relevance via reduced Aβ stress and downstream kinase/inflammation activation	Emphasises upstream disease stress modulation rather than direct tau binding	[56]
*Kaempferia* (methoxyflavones)	Integrated computational + multitarget screening	In silico target interaction plus anti-Aβ aggregation and cholinesterase inhibition	Provides a framework for multi-target rhizome-derived intervention; tau inference via network-level AD stress reduction	Needs tau endpoints to claim anti-tau action	[57]
*Zingiber* (6-gingerol/6-shogaol-enriched phytosomes)	Formulation + in vitro neuroprotection	Phytosome encapsulation improves cellular protection against oxidative neurotoxicity	Oxidative stress reduction can reduce kinase activation and proteostasis stress relevant to tau pathology	Formulation may be essential for feasible CNS exposure	[58]

**Table 2:** In vitro evidence for rhizome-derived bioactives relevant to tau nodes (direct and indirect).

Rhizome-Derived Agent	Model System	Exposure or Regimen	Tau-Relevant Nodes Interrogated	Main Findings (Brief)	Primary Source
Curcumin	Tau protein aggregation assays	In vitro biophysical assays	Tau fibrillisation or disaggregation	Inhibited tau aggregation and reported disruption of preformed tau filaments	[2]
Curcumin	SH-SY5Y + Aβ challenge	Pre-treatment paradigm (cell model)	Tau phosphorylation upstream signalling	Reduced Aβ-induced tau hyperphosphorylation via PTEN/AKT/GSK-3β signalling	[59]
Curcumin	SH-SY5Y + acrylamide stress	Pre-treatment paradigm (cell model)	Stress kinase → tau phosphorylation coupling	Suppressed PERK-eIF2α signalling with downstream suppression of abnormal tau phosphorylation	[60]
Novel curcumin derivatives	SH-SY5Y and primary neuronal cultures	In vitro screening	Tau oligomer pathway modulation	Curcumin derivatives reported to modulate tau oligomer aggregation pathways and reduce toxicity signatures	[61]
6-Gingerol (*Zingiber*)	Differentiated PC12 + Aβ1-42	40–300 μM pre-treatment, then Aβ exposure	AKT/GSK-3β axis (tau kinase node)	Increased p-AKT and inhibitory p-GSK-3β; reduced oxidative stress and apoptosis markers	[62]
ACA (*Alpinia*)	Differentiated PC12 + Aβ fragment	Time-limited exposure; pathway inhibitors used	Proteasome (UPS) support	Increased proteasome activity via cAMP/PKA-linked mechanism; improved viability under Aβ fragment stress	[28]
Methoxyflavones (*Kaempferia*)	Aβ aggregation assays; SH-SY5Y protection	Multi-assay pipeline	Aggregation suppression and neuronal resilience	Methoxyflavones inhibited Aβ aggregation, destabilised fibrils, and protected SH-SY5Y cells from Aβ toxicity	[57]
6-Gingerol/6-shogaol phytosomes (*Zingiber*)	Oxidative stress-induced neurotoxicity assays	Phytosome vs. non-encapsulated comparisons	Oxidative stress resilience (tau pressure reduction)	Formulated extracts protected against oxidative neurotoxicity, supporting formulation-first translational logic	[58]

**Table 3:** In vivo evidence (animal models) relevant to tauopathy and neuro–metabolic tau coupling.

Agent (Rhizome Origin)	Model	Dose or Duration	Tau Endpoint(s)	Main Outcomes	Primary Source
Curcumin	Scopolamine-induced AD-like rats	80 mg/kg oral gavage for 28 days	Brain and plasma phosphorylated tau epitopes; kinase regulators	Reduced tau hyperphosphorylation with changes in GSK-3β and CDK5/p25 signalling; behavioural improvement	[63]
Curcumin nanoparticles	HFD/STZ type 2 diabetes + neurodegeneration rats	CurNP 10 or 50 mg/kg oral daily for 6 weeks	Tau hyperphosphorylation in the hippocampus	Reduced tau hyperphosphorylation (strongest effects reported for specific nanoparticle dosing), with improved oxidative and inflammatory profiles	[71]
Curcumin (engineered exosome formulations)	Tau transgenic mice (tauP301S)	CUR-loaded constructs via IV delivery; multiple behavioural tests	Tau phosphorylation in brain tissue	Improved learning/memory behaviours and reduced tau phosphorylation; mitochondrial-protective mechanism emphasised	[53]
Curcumin derivative (Shiga-Y5)	rTg4510 tauopathy mice (P301L)	500 ppm in chow (~83 mg/kg/day) for 4 months	Tau accumulation (including insoluble fractions)	Reported inhibition of cognitive impairment and tau accumulation in a tauopathy model	[85]
Ginger extract (*Zingiber*)	ICV-STZ brain insulin resistance rats	300 mg/kg (reported extract regimen)	Indirect tau node: IRS, GSK-3β gene expression	Mitigated behavioural and histopathological changes; molecular shifts include IRS and GSK-3β changes	[54]
Ginger root extract (*Zingiber*)	AD-like rat behavioural dysfunction model	Dose-range design in the rat model	Tau is not the primary endpoint	Improved behavioural measures and reduced inflammatory/oxidative markers, supporting the upstream tau-pressure reduction concept	[86]
*Kaempferia parviflora* extract	Scopolamine-induced amnesic mice	Extract intervention in the behavioural paradigm	Tau not assessed	Memory improvement; integrated multi-target profile (AChE/BChE, Aβ aggregation)	[57]
ACA (*Alpinia*)	Senescence-accelerated context and cell-linked evidence	Mechanistic emphasis on AMPK and proteostasis	Tau was not assessed directly	Positions ACA as an AMPK-linked, proteostasis-relevant candidate, mechanistically compatible with tau clearance logic	[87]

**Table 4:** Clinical and human evidence (tau-relevant when available).

Intervention (Rhizome Origin)	Population and Design	Dose or Duration	Tau-Relevant Endpoints	Main Findings	Primary Source
Bioavailable curcumin (Theracurmin)	Middle-aged and older adults without dementia, randomised placebo-controlled trial	90 mg twice daily for 18 months	FDDNP-PET (binds amyloid and tau-related pathology), cognitive testing	Reported memory improvement and changes in PET signal consistent with reduced pathological binding	[88]
Curcumin C3 Complex	Mild-to-moderate AD; randomised double-blind placebo-controlled with extension	2 g/day or 4 g/day; 24 weeks (extension to 48)	CSF total tau and p-tau181; plasma Aβ; ADAS-Cog	Generally well tolerated with GI withdrawals; no differences in clinical or biomarker outcomes; low plasma curcumin levels	[73]
Curcumin formulation (Biocurcumax)	Community-dwelling older adults; randomised placebo-controlled double-blind	1500 mg/day for 12 months	Biomarkers not central; cognition tracked longitudinally	Small signal on in one cognitive measure; authors emphasised the need for biomarker-linked follow-up	[89]
Ginger extract (*Zingiber*)	Healthy middle-aged women; double-blind placebo-controlled randomised trial	400 mg or 800 mg daily; 2 months	No tau biomarkers	Improved attention/cognitive processing measures and ERP components in a dose-responsive pattern	[90]
*Alpinia galanga* extract (E-AG-01)	Healthy adults; randomised double-blind placebo-controlled crossover	Acute testing across visits	No tau biomarkers	Improved alertness and sustained attention metrics vs. placebo; relevance is symptomatic/cognitive rather than disease-modifying	[91]
Curcumin supplementation meta-analysis	Aggregated RCT evidence (mixed populations)	Dose/duration stratified	Typically not tau-specific	Reports overall cognitive effects with heterogeneity; highlights dose and duration as moderators	[92]

## Data Availability

No new data were created or analyzed in this study. Data sharing does not apply to this article.
